# Pelagic calcifiers face increased mortality and habitat loss with warming and ocean acidification

**DOI:** 10.1002/eap.2674

**Published:** 2022-07-27

**Authors:** Nina Bednaršek, Brendan R. Carter, Ryan M. McCabe, Richard A. Feely, Evan Howard, Francisco P. Chavez, Meredith Elliott, Jennifer L. Fisher, Jaime Jahncke, Zach Siegrist

**Affiliations:** ^1^ Marine Biological Station National Institute for Biology Ljubljana Slovenia; ^2^ Cooperative Institute for Marine Resources Studies Oregon State University Newport Oregon USA; ^3^ Cooperative Institute for Climate, Ocean, and Ecosystem Studies University of Washington Seattle Washington USA; ^4^ NOAA Pacific Marine Environmental Laboratory Seattle Washington USA; ^5^ Department of Geosciences Princeton University Princeton New Jersey USA; ^6^ Monterey Bay Aquarium Research Institute Moss Landing California USA; ^7^ Point Blue Conservation Science Petaluma California USA; ^8^ System Science Applications, Inc Renton Washington USA

**Keywords:** California Current Ecosystem, climate change, global data synthesis, habitat loss, habitat suitability modeling, multiple stressors, ocean acidification, pelagic calcifiers, pteropods, species distribution, warming

## Abstract

Global change is impacting the oceans in an unprecedented way, and multiple lines of evidence suggest that species distributions are changing in space and time. There is increasing evidence that multiple environmental stressors act together to constrain species habitat more than expected from warming alone. Here, we conducted a comprehensive study of how temperature and aragonite saturation state act together to limit *Limacina helicina*, globally distributed pteropods that are ecologically important pelagic calcifiers and an indicator species for ocean change. We co‐validated three different approaches to evaluate the impact of ocean warming and acidification (OWA) on the survival and distribution of this species in the California Current Ecosystem. First, we used colocated physical, chemical, and biological data from three large‐scale west coast cruises and regional time series; second, we conducted multifactorial experimental incubations to evaluate how OWA impacts pteropod survival; and third, we validated the relationships we found against global distributions of pteropods and carbonate chemistry. OWA experimental work revealed mortality increases under OWA, while regional habitat suitability indices and global distributions of *L. helicina* suggest that a multi‐stressor framework is essential for understanding pteropod distributions. In California Current Ecosystem habitats, where pteropods are living close to their thermal maximum already, additional warming and acidification through unabated fossil fuel emissions (RCP 8.5) are expected to dramatically reduce habitat suitability.

## INTRODUCTION

Human CO_2_ emissions have caused significant physical and biogeochemical alterations in the global oceans (Friedlingstein et al., 2019; Gattuso et al., 2015; Le Quéré et al., 2018), causing ocean warming, acidification and deoxygenation (Doney et al., 2020; Feely et al., 2004). Multiple lines of evidence suggest that these concurrent effects of global anthropogenic change will result in long‐term negative ecological consequences for marine organisms (Chan et al., 2017; Feely et al., 2008, 2016, 2018; Gruber et al., 2012; Turi et al., 2016).

Temperature and carbonate saturation state are two major drivers that shape physiological limits of marine calcifiers and define their vulnerability to ongoing ocean warming and acidification (OWA), with a growing body of evidence indicating that these changes may already be underway (Bednaršek et al., 2020; Fox et al., 2020; Osborne et al., 2020). However, to date, there are limited mechanism‐based risk assessments that integrate multiple lines of sensitivity and specific habitat requirements. Such studies are important not only to delineate potential habitat loss of pelagic calcifiers but also to quantify the risks of various representative carbon emission pathways (RCPs) and to contextualize potential conservation strategies. RCP8.5 assumes “no greenhouse gas mitigation” and RCP2.6 reflects “strong mitigation.” The latter scenario limits the increase in global mean surface temperature to below 2°C (relative to the reference period 1850–1900) and is therefore suitable for providing a first estimate for the consequences of keeping global warming to “well below 2°C, if not 1.5°C,” as stated in the Paris Agreement (Oppenheimer et al., 2015).

Among the calcifiers, pelagic gastropod communities, including pteropods, have been identified as particularly sensitive to global changes. Pteropods are globally distributed, with important ecological and biogeochemical roles in regional ecosystems such as the temperate habitats of the California Current Ecosystem (CCE; Bednaršek et al., 2014), and even more important roles in the subpolar and polar regions of the Arctic and the Southern Oceans (Bednaršek et al., 2012; González et al., 2018; Wang et al., 2017). These habitats are projected to experience higher rates of OWA than other areas (Siedlecki et al., 2021). Many experimental studies ascribe negative effects on pteropods to OWA (Bednaršek et al., 2017, 2018; Gardner et al., 2017; Hoshijima et al., 2017; Johnson & Hofmann, 2020; Lischka et al., 2011; Lischka & Riebesell, 2012). Among pteropods, *Limacina helicina* is the most dominant and ecologically important species in the northern hemisphere, with a widespread geographic distribution (Bednaršek et al., 2017). Studies have shown that *L. helicina* is negatively impacted by ocean acidification, while interactive stressors (e.g., OWA) can shape their cellular and physiological responses (Bednaršek et al., 2017, 2018; Lischka et al., 2011). However, currently there are no regional or global scale indices of environmental suitability available for pteropods that use multiple stressors to project biological responses.

Various environmental parameters shape organismal physiological tolerances, with temperature considered the dominant factor influencing the physiology and distribution of ectothermic organisms like *L. helicina* (Robinson et al., 2017; Wethey et al., 2011). However, the carbonate system chemistry can independently induce physiological stress through increased energy requirements to maintain homeostatic strategies, with uncompensated costs leading to the changes in the species' fitness, survival and population distribution. While some mechanisms driving the biological response related to a single stressor are better understood (McLaren, 1963; Smith & Teal, 1973) the combined effect of OWA has rarely been investigated, especially at the population level, underscoring the need for a better understanding of how multi‐stressor change influences marine calcifiers.

Habitat Suitability Indices (HSIs) are emerging tools to empirically predict the likelihood of species occurrences based on their biogeographical and macroecological patterns. So far, only a few HSIs have been developed specifically for zooplankton in regard to climate‐change‐related stressors, despite a clearly identified need to better characterize the impacts of ocean changes. This is in part because developing and testing empirical HSIs requires synoptic information about species distributions, their physiological traits, and physical–chemical habitat characterization. These measurements are seldom recorded together, thus limiting assessments of population impacts due to multiple stressors.

**FIGURE 1:**
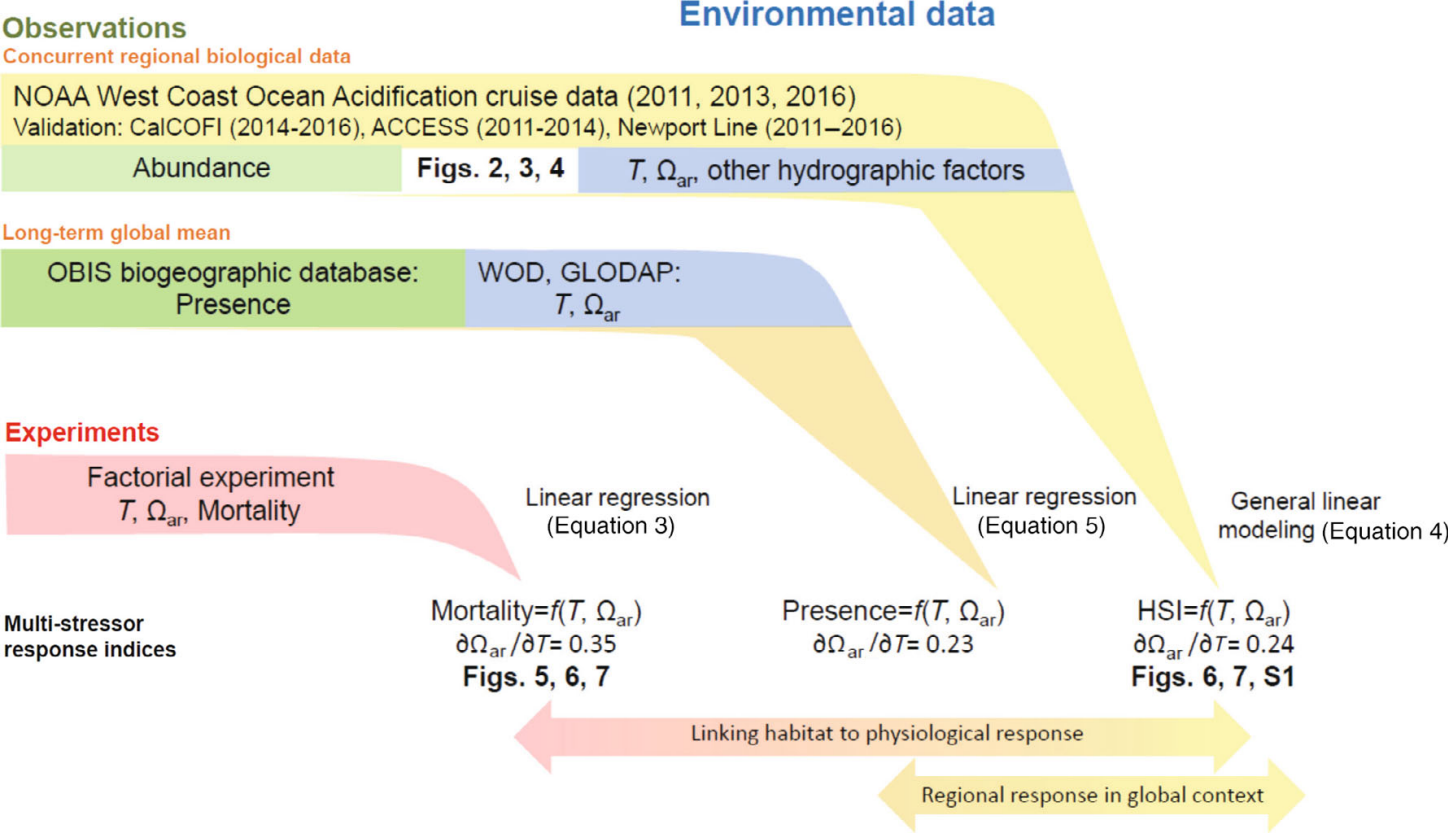
Schematic representation of the relationships between the data sets, variables, statistical analyses, and outputs presented in this study.

In this study, we compared concurrent field *L. helicina* observations and in situ physicochemical water properties. Specifically, we used three synoptic field surveys spanning 2011–2016, in combination with regionally colocated time‐series observations, to develop an empirical habitat suitability index (HSI) based on temperature (*T*) and the saturation state of aragonite (Ω_ar_). To test whether this empirical population‐level HSI is underpinned by, and adequately represents, pteropod sensitivity to the dual stressors (OWA), we conducted shipboard multifactorial experimental studies exploring the effects of OWA on *L. helicina*. Finally, we compared globally distributed *L. helicina* observations and climatological hydrographic information from the World Ocean Atlas (WOA; Garcia et al., 2018) and the Global Ocean Data Analysis Project gridded data product (GLODAP; Lauvset et al., 2016) to evaluate whether the physicochemical sensitivities and the HSI generated from our CCE analysis are broadly consistent with the known geographic and hydrographic distribution of this species. Relationships between the data sets, analyses, and statistical approaches presented in this work are schematically outlined in Figure 1. By combining global and regional observations, experiments and statistical modeling, this study represents the most comprehensive study thus far to explore how multiple drivers (OWA) influence *L. helicina*.

## MATERIALS AND METHODS

### Physicochemical and biological sampling

#### NOAA cruises

**FIGURE 2:**
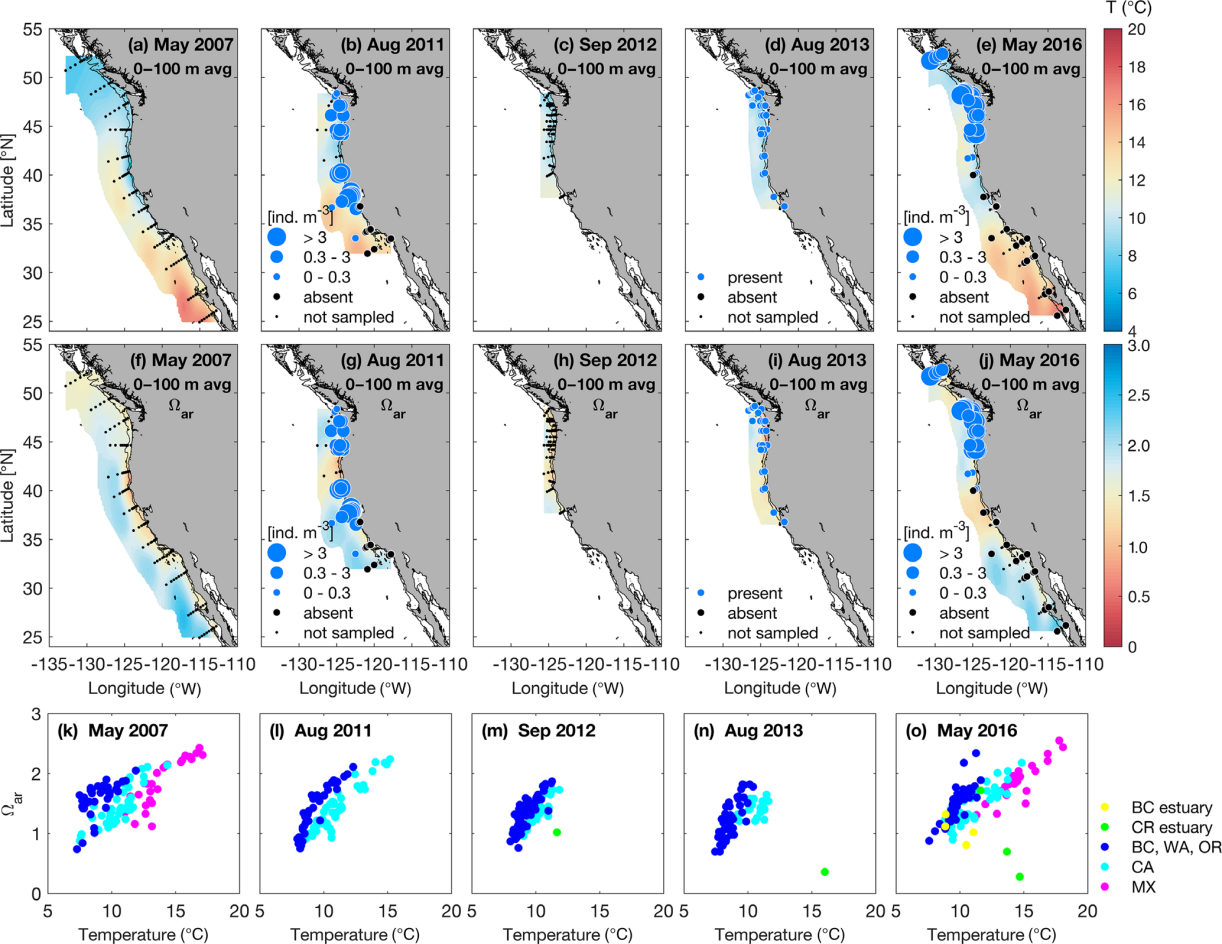
Physical and chemical observations captured strong hydrographic gradients of (a–e) temperature (*T*) and (f–j) aragonite saturation state (Ω_ar_) in the California Current Ecosystem (CCE). Abundances of the dominant pteropod species, *Limacina helicina*, are included for 2011 and 2016, and *L. helicina* presence/absence is included in 2013. (k–o) Linear relationship between temperature and Ω_ar_ averaged over 100 m across different geographic regions (BC, British Columbia; CR, Columbia River; WA, Washington; OR, Oregon; CA, California; MX, Mexico).

During the NOAA West Coast Ocean Acidification (WCOA) cruises in 2007, 2011, 2012, 2013 and 2016, vertical profiles of temperature (*T*), salinity (*S*), macronutrients, oxygen, chlorophyll *a*, dissolved inorganic carbon (DIC), total alkalinity (TA), and pH were sampled along 17 cross‐shelf transects, and *p*CO_2_ and aragonite saturation state (Ω_ar_) were calculated following the methods described by Feely et al. (2016). Biological sampling was also conducted in 2011, 2013, and 2016 (Figure 2). Pteropods were generally collected during nighttime sampling at 48 stations, using 333‐μm mesh Bongo nets with 30–45‐minute oblique tows from 100 m depth to the surface (Bednaršek et al., 2017).

#### Regional time series

**FIGURE 3:**
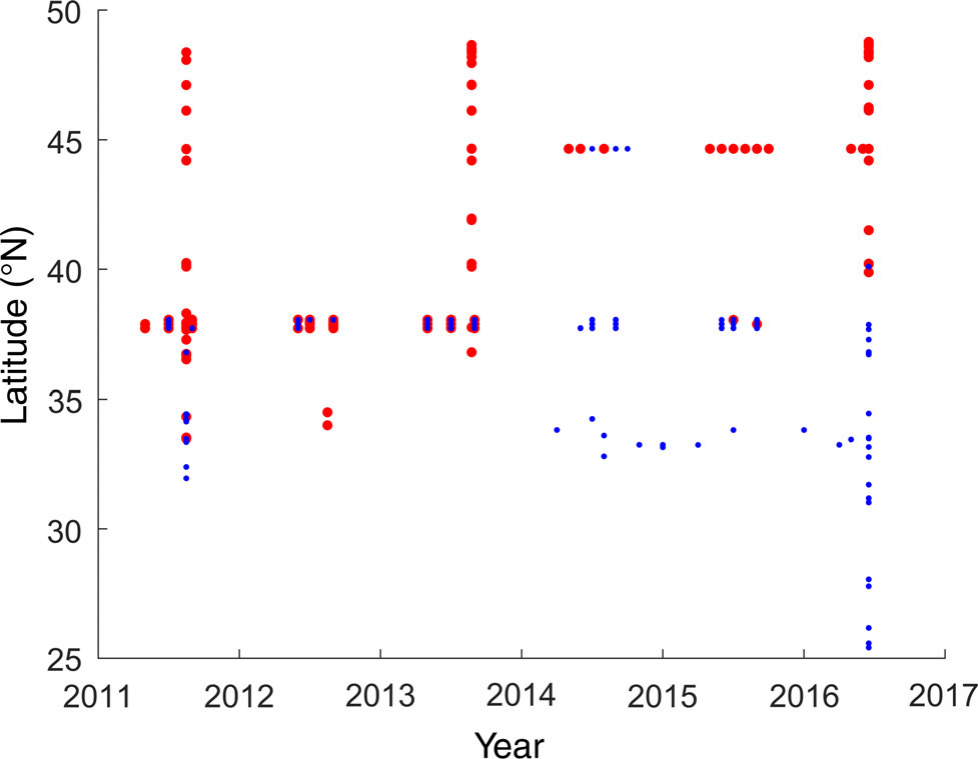
Compilation of large‐scale and regional presence/absence data for *Limacina helicina* from 2011 to 2016 across latitudinal scales from 25° to 50° N. Blue dots denote *L. helicina* presence, red dots denote their absence.

**FIGURE 4:**
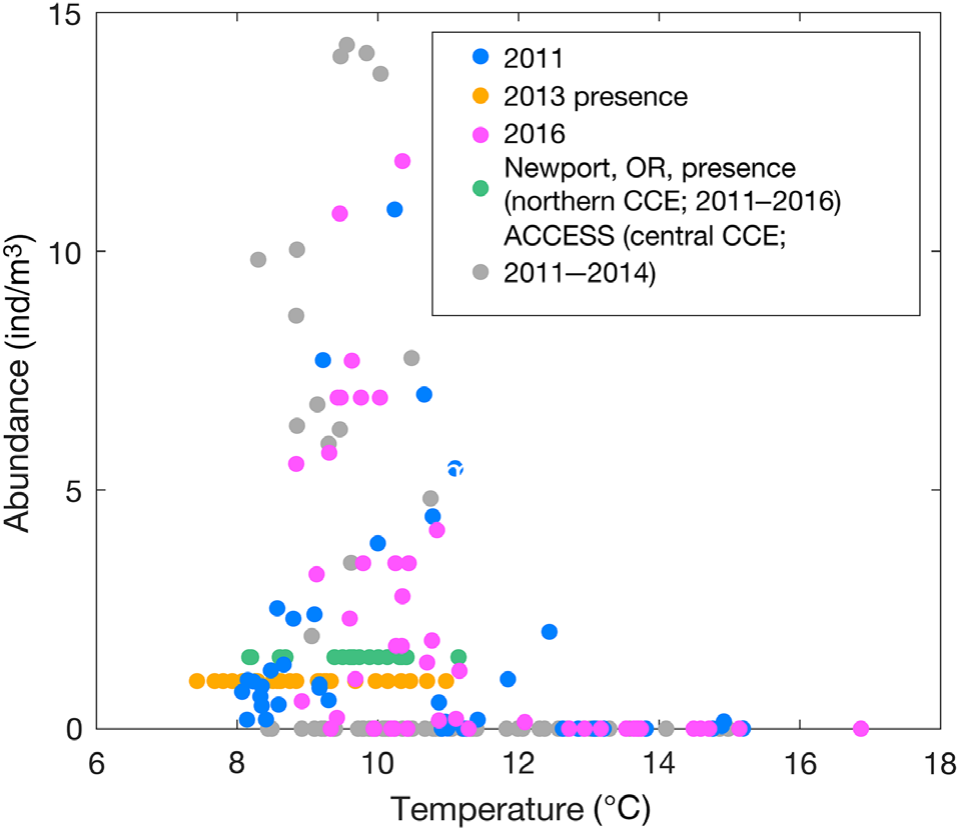
Abundance distribution of *Limacina helicina* related to temperature integrated over the upper 100 m. Many samples contained >15 individuals/m^2^, however, none of those were associated with average temperatures >11.6°C.

We supplemented the synoptic cruise data sets with three regional time series spanning different periods (https://www.ncei.noaa.gov/access/ocean‐carbon‐data‐system/oceans/Coastal/WCOA.html.). These time series include *L. helicina* presence or absence observations (Figure 3) along with local hydrographic data (Figure 4). In the southern CCE, pteropod presence data for 2014 to 2016 was obtained from the CalCOFI program zooplankton database using vertically towed nets (202 μm mesh PRPOOS) spanning the surface to 210 m, and laterally towed MOCNESS nets for vertically stratified sampling. Central CCE pteropod data between 2011 and 2014 was obtained from the Applied California Current Ecosystem Studies (ACCESS) cruises off San Francisco, CA, USA. Zooplankton was sampled using a 1 m diameter hoop net (333 μm mesh) towed obliquely for 10 minutes from 50 m to the surface. Northern CCE data spanning 2011 to 2016 were obtained from the Newport Hydrographic Line (NH25) off central Oregon, at a station located 40 km offshore over 200‐m bathymetric depth. These samples were collected using a vertically towed 0.5 m diameter net (202 μm mesh) between 100 m and the surface. Because the sampling methodologies vary between these time series, we minimized potential biases in abundance between the data sets by restricting our analysis to presence/absence‐only data.

Paired hydrographic data were available from the Newport and ACCESS time‐series stations, though pH and TA were not sampled and are instead estimated in this work using locally interpolated regressions based on temperature, salinity, and oxygen concentration (Carter et al., 2018) with pH adjustments for OA applied. The synthetic Ω_ar_ values estimated at the ACCESS and Newport sites were therefore used only for validation statistics presented in Appendix S1 and do not contribute to training HSI models.

#### Global databases

Globally distributed *L. helicina* observations were obtained from the Ocean Biodiversity Information System (OBIS; https://obis.org/taxon/140223). This database includes primarily polar and subpolar presence data (generally not absence) and does not include any CCE data; we used these independent data to test whether the relationships derived from the CCE are plausible and consistent with environmental sensitivities over the broader species range.

The maximum and minimum depths associated with each observation encompassed a large hydrographic range. Concurrent hydrographic observations were not available for these OBIS occurrences, so we instead relate these observations to climatological mean ocean properties, which can be useful metrics for assessing the long‐term mean environmental sensitivities of species biogeography (e.g., Deutsch et al., 2020; Righetti et al., 2019). Each pteropod observation from OBIS is paired with the annual climatological temperature within the standard WOA hydrographic grid cell encompassing the trawl location and depth (WOA version 2018, Garcia et al., 2018). Ω_ar_ for the same coordinates are obtained from the GLODAP gridded annual climatology (Lauvset et al., 2016). We use annual climatologies because carbonate system chemical observations are generally too sparse to derive robust seasonal or monthly Ω_ar_ climatologies below the ocean surface. Moreover, the relevant OBIS observations for the analysis presented are well below the seasonal thermocline; thus, deviations from climatological conditions are generally small and well bounded (Boyer et al., 2018; Roemmich & Gilson, 2009). Additional details of OBIS data processing and the pairing of biological and hydrographic data are included in the Appendix S1.

### Pteropod dual‐stressor incubation experiments

To determine the combined effects of thermal stress and low‐Ω_ar_ conditions on *L. helicina* survival, we conducted full‐factorial experiments onboard the WCOA 2016 cruise. Pteropods were examined for mechanical damage, and ~20 intact and actively swimming adults were placed in a flow‐through experimental conditions. Temperature was controlled using recirculating water carboys with thermal heaters in the mixing (pre‐exposure) chambers. Gas with fixed partial pressures of CO_2_ (*p*CO_2_) was continuously added to the treatments, and the seawater *p*CO_2_ was checked for consistent pH measurements using Durafet probes calibrated at 11°C with pH‐certified Tris buffer. We placed captured pteropods in shipboard flow‐through aquaria with water pre‐acclimatized to targeted CO_2_ levels within ±5% of 400 (control) and 1200 μatm (treatment) at 10°C. This corresponded to Ω_ar_ levels ranging from ~0.94 to ~2.2, and two temperature levels of ~9°C and ~13.5°C (Appendix S1: Table S1), representative of the ranges observed in the upper 100 m of the water column (Appendix S1: Table S1; Appendix S1: Table S2). For validation purposes, total alkalinity and dissolved inorganic carbon were measured at the beginning and the end of the experiments. In addition, discrete pH, oxygen, and temperature were measured daily to confirm the sensors were performing as expected.

We maintained pteropods (a total of 480 individuals; Appendix S1: Table S2) from 10 different stations for 1 week to investigate their survival at each treatment level. Twenty adults were kept in 4‐L carboys, with six replicates for each condition. The individuals were fed one spike of 50 μl/L Shellfish Diet 1800 (Reed Mariculture), a commercially prepared mix of four marine microalgae (*Isochrysis* sp, *Pavlova* sp., *Thalossiosira weissflogii*, and *Tetraselmis* sp.; >50,000 cells/ml). Pteropod survival was determined under a light microscope, based on the presence of individual heartbeat and movement. To test for differences in survival among treatments, we conducted two‐way mixed‐model ANOVA analyses. We also note that oxygen was maintained near atmospheric equilibrium (slightly supersaturated) at all times.

### Habitat suitability index model development and validation for *L. helicina*

Habitat suitability indices (HSIs) can be used to identify the environmental conditions in which an organism is typically found. We derived an HSI for the CCE based on WCOA cruise data. Specifically, we used generalized linear modeling, a statistically based empirical modeling approach. In this section, we review the construction of the HSI and its validation.

The HSI yields numbers between 0 and 1 with 0 indicating predicted absence and 1 indicating predicted presence. Numbers between 0 and 1 indicate probability of finding *L. helicina* in a net tow using the same tow protocols used on the WCOA research cruises.

The HSI model is derived from a linear function (LF, ranging from −∞ to ∞) based on seawater properties to estimate the probability of *L. helicina* presence. The LF is the sum of the products of *n* linear regression coefficients (α_*i*_ terms) and their associated *n* measured seawater predictor property values (*P* terms) plus a constant term α_0_

(1) $$\mathrm{LF}=\alpha_{0}+\sum_{i=1}^{n}P_{i}\alpha_{i}.$$

The HSI is calculated from the LF using the logistic function to transform the results from an infinite scale to an index value between 0 and 1

(2) $$\mathrm{HSI}=\frac{e^{\mathrm{LF}}}{e^{\mathrm{LF}}+1}.$$

The α_*i*_ regression coefficients in Equation 1 are fit using the stepwiseglm routine (Matlab v2014b). This routine iteratively adds or removes model parameters based on whether additional terms improve the model fit (χ^2^ test at 95% confidence). Numerous combinations of predictors and depth surfaces were tested and evaluated based on the skill with which the models reproduced data withheld during training. These trials and the validation metrics are described in greater detail in Appendix S1. The validation metrics for the model based on the properties of interest (*T* and Ω_ar_) equaled or exceeded metrics for all alternative empirical models based on other parameter combinations. After similar testing of depth levels, the property averages over the top 0–100 m were selected and used; this had the best predictive power across models for the WCOA data. This depth range also corresponded to the vertical extent predominantly sampled for *L. helicina* with plankton tows, which contains 90% of *L. helicina* observations globally (OBIS) and is the most frequently reported vertical habitat for this organism (Bednaršek et al., 2012; Lalli & Gilmer, 1989).

The HSI models were assessed using a model trained only with WCOA observations from 2011 to reconstruct observations from 2013 to 2016 and using a 2016‐only model to reconstruct observations from 2011 to 2013. Cruise data from 2013 were not used to train either of the models used for validation because only pteropod presence, and not absence, was recorded.

Skill improvement (SI) was our primary model validation metric (Appendix S1: Section S1; Appendix S1: Table S3; Appendix S1: Table S4), describing how much more accurate the model is than a constant probability model. In this constant probability model, the probability of presence is set equal to the frequency that pteropods are found across all net tows irrespective of environmental conditions (e.g., 56%–65% depending on year). SI is expressed as a percentage of the difference between the skills of a constant model and a hypothetical “perfect infinite‐confidence model” that only makes correct predictions. The SI is 0% for the constant probability model and 100% for a perfect model. Higher SI values imply that the HSI model is increasingly skillful compared to the constant probability model that assumes environmental conditions do not matter and that the probability of finding pteropods is identical everywhere.

### Comparison of regional to global *L. helicina* records

We compare global presence observations of *L. helicina* to climatological temperature and Ω_ar_ values in the same locations where the pteropods were observed. Specifically, we binned the presence by 1°C *T* and 0.2 Ω_ar_ increments. The minimum Ω_ar_ concurrent with pteropod observations at each temperature defines an empirical threshold between pteropod presence and their lack of over the global range of observations. For comparison with the HSIs from the CCE, we fit a least‐squares regression to these minimum Ω_ar_ between 8°C and 13°C; this is the overlapping temperature range between the predominantly polar OBIS data and the WCOA CCE data (Appendix S1: Section S2). We did not generate an HSI from the global data directly because, unlike the WCOA cruise data, the OBIS database generally lacks abundance and absence information and reflects varied sampling approaches.

## RESULTS

### Spatial and temporal changes in pteropod distribution in the CCE

Using the coastwide *L. helicina* data set from 2011, 2013, and 2016 over the CCE, it is clear that the spatial and temporal distribution of *L. helicina* is wide ranging in the spring and summer seasons (Figures 2 and 3). However, *L. helicina* presence was temperature dependent, showing a unimodal abundance distribution when related to 100‐m averaged temperature. The realized thermal habitat window for this species (averaged over the upper 100 m) spanned ~8–13°C, with peak abundances around 10°C (Figure 4). This temperature distribution occurred consistently over several years regardless of seasonal and interannual variability, suggesting that these ranges are representative of the natural envelope of thermal conditions that defined suitable habitat for *L. helicina* in the CCE. When the temperature in the CCE increased above 15°C, *L. helicina* was largely absent, indicating a relatively stark upper thermal limit. With respect to Ω_ar_, pteropods occupied conditions in the upper 100 m that were generally supersaturated with respect to Ω_ar_ (Ω_ar_ > 1), and were only occasionally found in Ω_ar_ < 1 conditions at colder temperatures.

### Experiments identify multiple stressor impacts on survival

**FIGURE 5:**
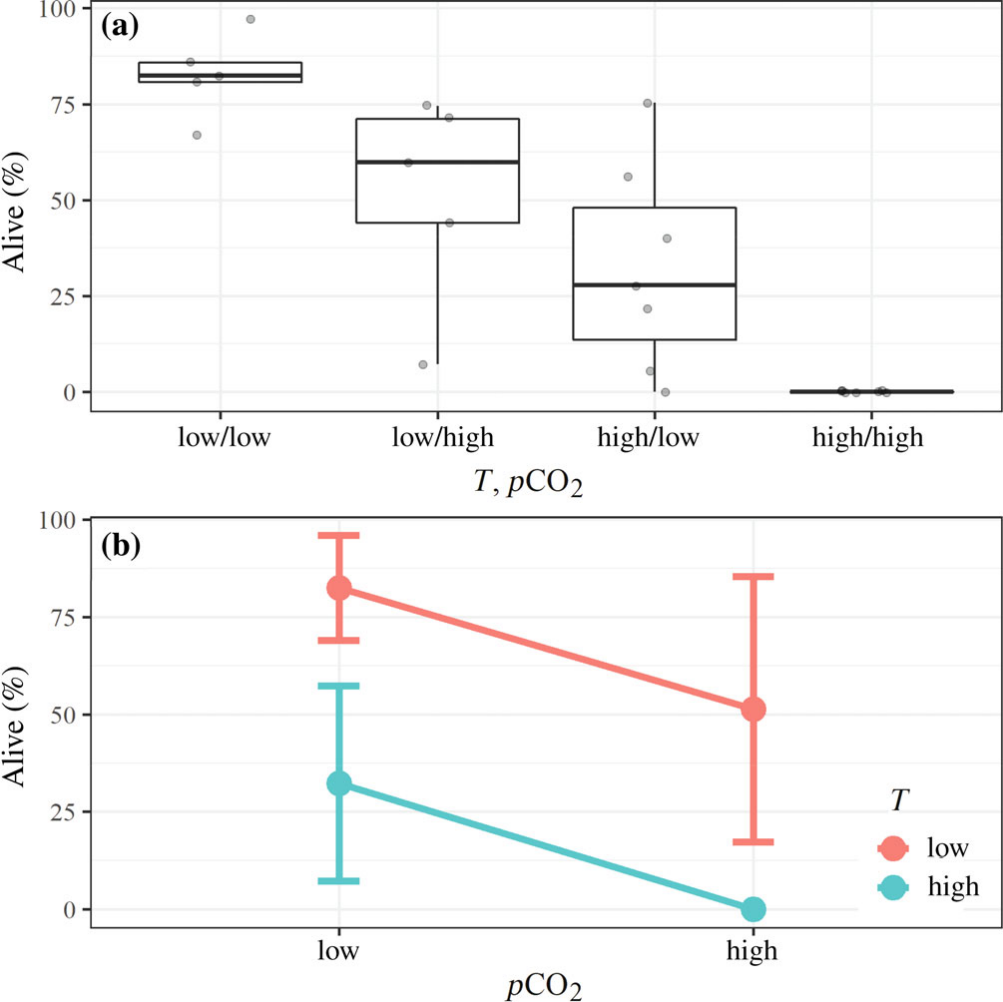
(a) Full‐factorial experiments included temperature and low Ω_ar_ on *Limacina helicina* (*N* = 480) using control (low *T*/low CO_2_), single stressor (low *T*/high CO_2_; high *T*/low CO_2_), or double stressor (high *T*/high CO_2,_ Appendix S1: Table S1; Appendix S1: Table S2). Data was analyzed using two‐way ANOVA and the results with standard deviation show 50% mortality due to thermal stress, 30% mortality due to high CO_2_/low Ω_ar_ conditions, and 100% mortality due to the combined stress. Box plot components are median (dark black line), interquartile range (light black lines), and full data range (whiskers). (b) The additive relationship is observed between survival (%) and combined stress of ocean warming and acidification (OWA) both at two different stressor levels (low/high *T*; low/ high OWA). Shown are the mean and standard deviation with 95% confidence interval.

Survival (±SD) under control conditions (low *T*/low CO_2_) was compared to survival under single (high *T*/low CO_2_; low *T*/high CO_2_) and interactive (high *T*/high CO_2_) stressor treatments (Figure 5) using ANOVA testing (Appendix S1: Table S2). Survival was greatest under control conditions (83% ± 9%), and significantly declined under exposure to a single stressor to 36% ± 25% (high *T*/low CO_2_) and 52% ± 26% (low *T*/high CO_2_; Figure 5a). Complete mortality was observed when the two stressors were combined (high *T*/high CO_2_). To estimate mortality, CO_2_ experimental treatment values were converted to Ω_ar_ values (Appendix S1: Table S1; Appendix S1: Table S2). Over the observed property ranges, both parameters were significantly related to mortality (Appendix S1: Table S2), with high temperature as a primary and low Ω_ar_ as a secondary driver for *L. helicina* mortality. Due to the complete mortality observed in the two‐stressor treatments, the possibility that the effects were synergistic rather than additive (Figure 5b) cannot be ruled out.

Additional analyses using a linear regression model (*R* ^2^ = 0.78, *F* = 58.5 with df = 33 numerator and df = 3 denominator, *p* < 0.001 for a null hypothesis of no trend) of all experimental trial replicate data produced Equation (3):

(3) $$\text{Percent mortality}=-19.4+11.5T-32.{\text{7Ω}}_{\mathrm{ar}}.$$

### Empirical HSI predicts presence/absence from seawater properties

The empirical HSI with *T* and Ω_ar_ was based on the WCOA observations, with the linear function for the empirical HSI model fit to the WCOA observations as follows:

(4) $$\mathrm{LF}=13.49-2.475T+10.10\Omega_{\mathrm{ar}}.$$

**FIGURE 6:**
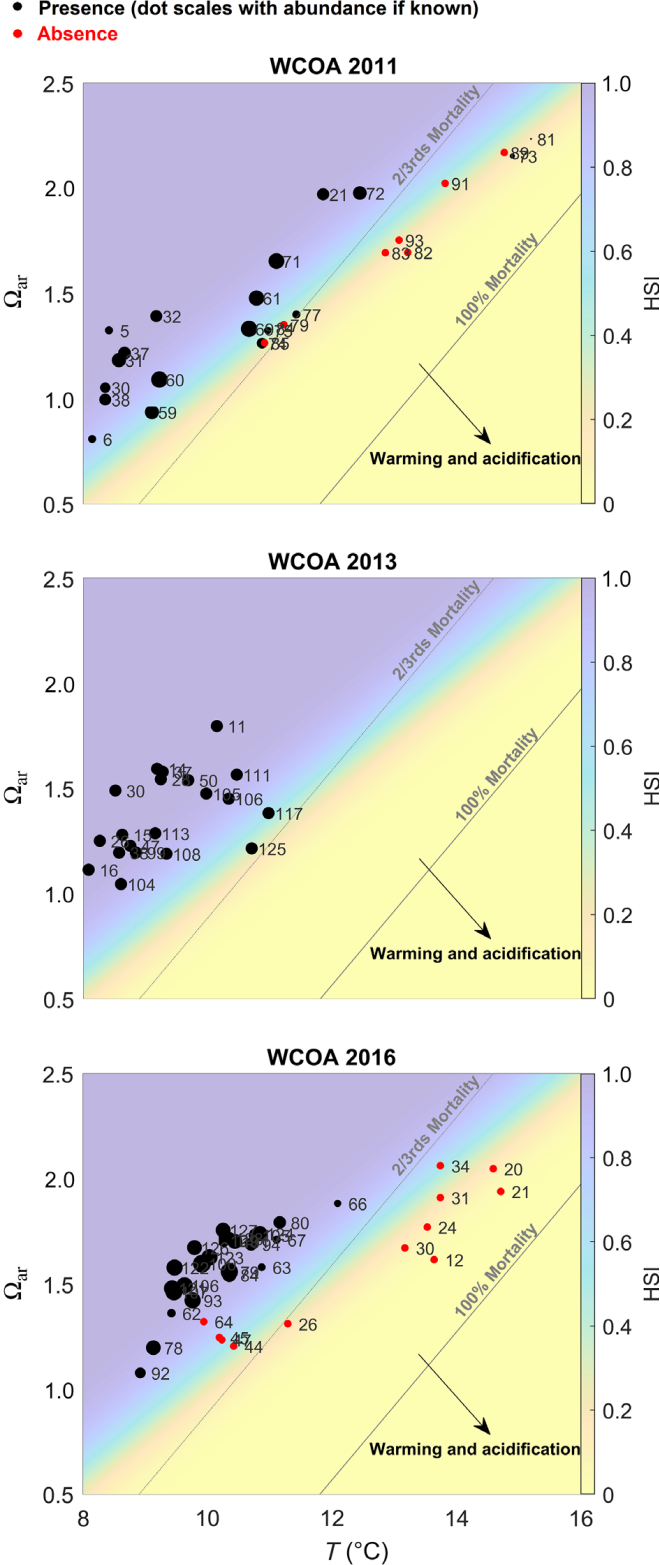
Conceptual *T*−Ω_ar_ diagram showing habitat compression based on temperature and Ω_ar_ as two stressors. Dots indicate presence (black dots) and absence (red dots) observed over 3 years (2011, 2013, 2016) of West Coast Ocean Acidification (WCOA) cruises with the numbers indicating the station numbers. The background color indicates the empirical Habitat Suitability Indices (HSI) values for each combination, while the dashed line shows the *T* and Ω_ar_ combinations at which the mechanistic HSI based on experimental incubations suggests two‐thirds experimental mortality. The proximity of the band at ~0.5 empirical HSI value to the dashed line implies a strong similarity between the two relationships, suggesting similar underlying mechanisms account for both. The HSIs show pteropods to be more abundant in the upper left corner, while more likely to be absent in the unsuitable habitats in the lower right.

The LF and the resulting HSI were plotted alongside estimated percent mortality from *T* and Ω_ar_ (Figure 6). Spatial HSI distributions (Appendix S1: Figure S1) closely reflect the spatial patterns in the physicochemical properties from which they were derived (Figure 1).

Coefficients for this and several alternative HSIs we discuss are given in Appendix S1: Table S3. We estimated the overall SI for our model as 55%, meaning this model significantly outperforms a constant probability model for reconstructing *L. helicina* distributions based on the WCOA data. We estimated this overall model SI by averaging the values for the related models obtained from 2016 (tested on 2011 and 2013 data) and 2011 (tested on 2013 and 2016 data). The SI for the 2011‐trained model is 70%, while the SI for the 2016‐trained model is 39%.

Evaluating the HSI with the ACCESS and Newport presence/absence time‐series observations is more difficult for two key reasons: first, these sites were observed year‐round, whereas our HSI is constructed solely from observations in the spring and summer and, second, concurrent carbonate chemistry measurements needed to evaluate the HSI are not available from those records. Therefore, (as noted in *Regional time series*) we assess the HSI for the time series using synthetic carbonate chemistry data estimated using regional, empirical relationships between Ω_ar_ and available *T*, *S*, and O_2_ (Carter et al., 2018); these relationships are also calibrated to spring and summer data. Because both our model and the estimated data are derived from spring and summer observations, the HSI can only be validated against the subset of these time series from April through September: the resulting SI scores are 22% at ACCESS and 38% at the Newport Hydrographic line for these months. The positive SI scores indicate the HSI also improves upon a constant probability model at these locations during the spring and summer months.

### Global observations have similar relationships to HSIs from the CCE

**FIGURE 7:**
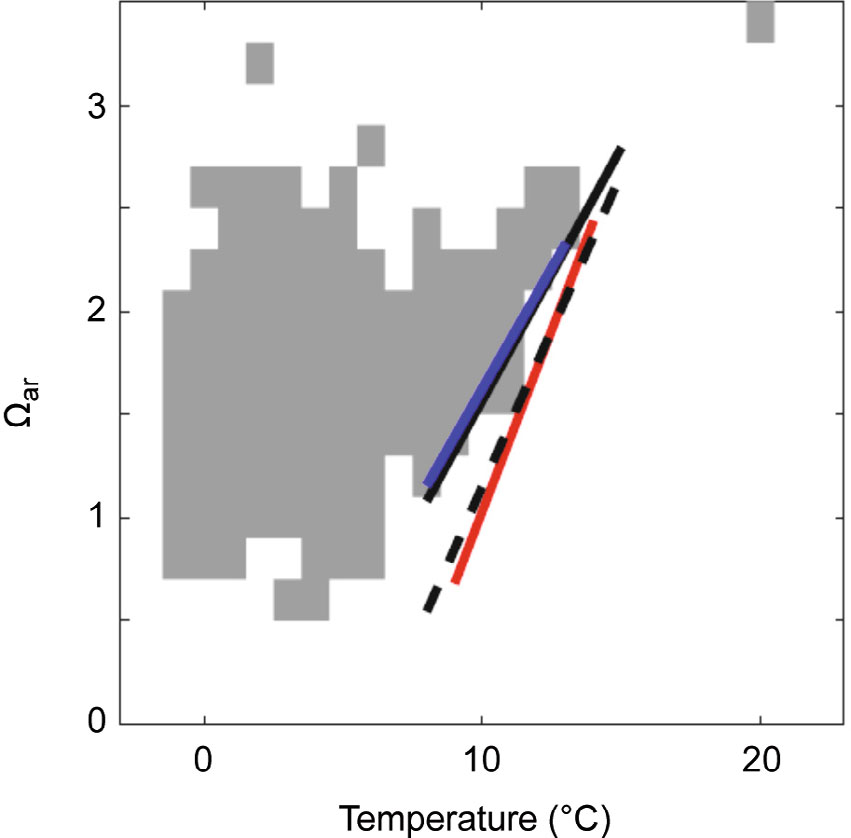
Globally distributed *Limacina helicina* presence (from OBIS, shaded gray) with respect to annual mean temperature (WOA) and Ω_ar_ (Global Ocean Data Analysis Project [GLODAP]) at the maximum depth of the vertical range reported for each observation. The blue line is the linear fit to the minimum observed Ω_ar_ over the range of 8–13°C (ΔΩ_ar_/Δ*T* = 0.23, *R* ^2^ = 0.87, *p* = 0.02 for null hypothesis of no correlation); this represents the overlapping temperature range for the polar and subpolar OBIS observations (no data from California Current Ecosystem [CCE]) and subtropical West Coast Ocean Acidification (WCOA) observations (from CCE). The solid black line is the empirical Habitat Suitability Indices (HSI) = 0.99 from the WCOA cruises (to approximate a presence/absence threshold for comparison with the OBIS data). The solid red line is the experimental mortality relationship equivalent to HSI = 0.99. The dashed black line is the empirical pteropod relationship from Bednaršek et al. (2018) with abundance = 0 (to approximate a presence/absence threshold; any non‐zero abundance leads to presence).

Globally and in a long‐term average sense, *L. helicina* were predominantly observed where low temperature and high Ω_ar_ occurred concurrently (Figure 7). Equations (3) and (4) both indicate that reduced survival from temperature increases can be partially offset with Ω_ar_ increases. This suggests that there is a temperature‐dependent threshold of Ω_ar_ below which *L. helicina* were more likely to be absent than present (defined by setting LF equal to zero in Equation 4). OBIS and climatological hydrographic data are similarly related: over the overlapping 8–13°C range between the high latitude OBIS data and the CCE data, the minimum Ω_ar_ at which the pteropods were found (Ω_ar,min_) can be represented as

(5) $$\Omega_{\mathrm{ar},\min}=0.23T-0.72.$$

The 95% confidence intervals on the coefficients were 0.07 to 0.40 for the slope (*r* ^2^ = 0.87, *p* = 0.02 for null hypothesis of 0) and −2.46 to 1.01 for the intercept (*p* = 0.28 for null hypothesis of 0). The global OBIS database covered a wider range of occupied *T* and Ω_ar_ environmental conditions than the WCOA observations (Figure 6); over this broader range, the temperature‐dependence of Ω_ar,min_ may be nonlinear. For example, at lower temperatures the apparent Ω_ar,min_ decreased less per degree of cooling than over the 8–13°C range. This response was consistent with Arrhenius‐like temperature dependence of physiological rates (as in temperature‐dependent hypoxia; Deutsch et al., 2020). However, other chemical, ecological, or biogeographic constraints could mediate this relationship, so more experimental evidence is required to confirm whether there is actually a nonlinear temperature dependence of acidification vulnerability grounded in organismal physiology.

Based on the global OBIS observations, *L. helicina* is widely distributed in the Northern Hemisphere oceans. These observations suggest that this species is a “thermal generalist,” for which the specific temperature range reflects the hydrographic conditions in different parts of its geographic range. As in the CCE, *L. helicina* was not commonly observed above temperatures of 13°C, though there was very little sampling of subtropical waters represented in the OBIS database. With respect to Ω_ar_, the global OBIS data set demonstrated that pteropods mostly occupy the habitats characterized by annual mean Ω_ar_ > 1_,_ however there were several observations where they occupied very low‐Ω_ar_ habitats, down to approximately Ω_ar_ = 0.6, with this always corresponding to the lowest temperature observations.

### Agreement between the empirical and experimental results

We compared experimentally derived mortality to the empirically derived HSI. To do this, we first reduced both relationships to lines separating more favorable and unfavorable conditions. For mortality, we set Equation (3) equal to 66%. For the HSI, we set Equation (4) equal to 0, which produces the line corresponding to conditions in which the HSI equals 0.5 (i.e., where there is equal likelihood of *L. helicina* presence and absence). Generally, at lower *T* and higher Ω_ar_ values (above those lines, plotted on Figure 5) our analyses suggest that conditions are more favorable and predict higher probability of presence and lower mortality. The choice of 66% versus any other constant probability (e.g., 0%, 50%, or 100%) only affects the intercept of these equations, and 66% was chosen so that these two lines would have a similar mean value across the CCE. We note that this mortality equation can serve as an LF for an HSI variant based on the experimental results (Appendix S1: Table S3) that has comparably strong validation statistics to the empirical HSI.

Importantly, these functions all generated broadly coherent habitat suitability thresholds in geographic space and in property–property space (Figures 6 and 7), and the slopes of all three relationships were remarkably similar: ∂Ω_ar_/∂*T* = 0.35 (the experimental relationship from Equation 3), 0.24 (the empirical relationship for the CCE from Equation 4), and 0.23 (the global empirical relationship from Equation 5). Similarly, the empirical CCE equation based on the observational data of Bednaršek et al. (2018) solved for a constant pteropod abundance leads to ∂Ω_ar_/∂*T* = 0.30. The comparability of all four of these relationships suggests that population‐scale presence and absence (the empirical results) were potentially linked to stressor‐induced mortality (the experimental results), and that the resulting population scale habitat constraints were consistent across a broad range of environmental conditions.

In summary, high *T* and low Ω_ar_ values correspond to high mortality and absence for *L. helicina* (bottom right of Figures 6 and 7), whereas high survival and presence were expected in colder and more saturated seawater (upper left of both figures). In the CCE, observations fell near HSI = 0.5 at both low and high temperatures because water in the CCE was largely a mixture of cold, low‐Ω_ar_ upwelled water (lower left of Figure 6) and warmer, higher‐Ω_ar_ surface and offshore water (upper right of Figure 6). Because the minimum Ω_ar_, below which pteropods were rarely observed, varied as a function of temperature, no constant thresholds in *T* or Ω_ar_ alone were sufficient to explain the observations: instead, a multi‐stressor framework is required. The empirical and experimental HSI metrics successfully capture these patterns of pteropod presence and absence with respect to both variables.

## DISCUSSION

Global climate change with the predicted shifts in the mean state and frequency and intensity of extremes of temperature and carbonate system parameters may play important roles in species distributions and habitat suitability (Oliver et al., 2018). In this study, we used three complimentary approaches to investigate the sensitivity of pelagic calcifier *Limacina helicina* to OWA. First, experimental incubations suggested that *T* and Ω_ar_ directly influenced organismal mortality. This agrees with the prior finding that low Ω_ar_ is a significant compounding factor on temperature for *L. helicina* mortality (Lischka et al., 2011). Furthermore, our study and the study by Lischka et al. (2011) found very similar results for the individual changes in survival with temperature increases and Ω_ar_ decreases as individual stressors, with 50% and 20%–30% explained variability, respectively. Second, regional HSI modeling showed these same properties strongly correlate with *L. helicina* distributions across multiple years, which suggests that both parameters affect habitat suitability in the CCE as well as in the laboratory. Third, the global, long‐term mean spatial alignment of hydrographic conditions and organism distributions was consistent with the regional CCE relationships over similar environmental conditions. This finding is notable given the non‐overlapping geographic coverage of the data sets and the differing ecosystems and environmental conditions considered, aside from *T* and Ω_ar_. This agreement, in the laboratory, regionally, and globally, is evidence in support of the key idea that *L. helicina* has interdependent and physiologically mediated sensitivities to temperature and aragonite saturation state.

The sensitivities we found by considering (primarily) spatial variability have a corollary in time. For each 1°C of warming, the minimum Ω_ar_ that *L. helicina* can tolerate in the CCE is increased by roughly 0.3. This relationship is consistent across the experimental and empirical work presented here. With concurrent OWA, we expect the distribution of this species to contract to a greater degree than implied by its thermal tolerance window alone. Conversely, climate variability or seasonal changes that result in cooling may allow the pteropods to tolerate lower Ω_ar_, which could expand habitat options.

*Limacina helicina* observations in the global OBIS database appeared to be well bounded by conditions at the bottom of reported depth ranges, but the maximum depth of *L. helicina* and concurrent hydrographic conditions were not readily obtainable from the WCOA data sets. Thus, it is possible that the predictive power of the HSI could be improved with a more precise identification of environmental thresholds associated with the pteropods' vertical ranges. More broadly, the versatility and applicability of the HSI may benefit from co‐located chemical and biological observations in other seasons and locations. Similarly, a better understanding of how the frequency and duration of stressful conditions impact *L. helicina* responses at various life stages could improve predictability of habitat occupancy.

Our results suggest that a multi‐stressor framework may be essential for understanding the responses of *L. helicina* to environmental stressors currently, as well as for future projections due to climate change. However, many studies that investigated long‐term pteropod population‐level responses have only taken a single driver into account (e.g., Howes et al., 2015; Thibodeau et al., 2019), but no explicit OA effects on the pteropod abundances. As demonstrated from our results, single parameter analyses are likely insufficient for explaining population level responses; multiple parameter frameworks have previously been demonstrated to hold greater explanatory power in some instances (e.g., Beare et al., 2013; Beaugrand et al., 2013; Head & Pepin, 2010). While these studies recognized temperature to be a major driver, we also observe that these results may apply only to the highest temperatures at which *L. helicina* are observed. We warn that acidification can have simultaneous effects at lower temperatures (sensu Lischka et al., 2011), where the effects may not cause immediate mortality but appear to be sufficiently large to exclude pteropods from potential habitat. If generalizable beyond the CCE, this is particularly concerning given that the high latitude environments where *L. helicina* are most commonly observed also have the lowest and most rapidly degrading carbonate system buffering capacity (Jiang et al., 2019). A caveat to our interpretation is that vulnerability to the two stressors may be nonlinearly related over the full range of hydrographic conditions that *L. helicina* inhabits. Qualitatively, the *T–*Ω_ar_ slope between suitable and non‐suitable habitat increases with temperature (Figure 7). This observation hints that further investigation is needed into the mechanisms driving temperature‐dependent acidification vulnerability outside of the examined conditions in this study. The linear relationships we report here are applicable to typical conditions within the CCE, but extreme events and climate‐driven shifts to novel conditions may require explicit consideration of nonlinear sensitivities to predict species distribution shifts.

With regards to future habitat conditions for *L. helicina* in the CCE, we consider two specific climate change scenarios; Representative Concentration Pathway (RCP) 8.5, which assumes “no greenhouse gas mitigation,” and RCP 2.6, which assumes “strong mitigation.” High anthropogenic CO_2_ concentrations in RCP8.5 will warm much of the ocean by 2°C or more. Based on the sensitivities in this work (over 8°–14°C), Ω_ar_ would have to increase by 0.5–0.7 on average to maintain the present extent of suitable habitat. In that regard, RCP2.6 suggests greater preservation of habitat than RCP8.5, and thus, lower risk for *L. helicina* populations. However, Ω_ar_ is projected to decrease concurrently in both scenarios because of OA. In the absence of strong carbon dioxide removal efforts, even the most stringent temperature mitigation scenarios are likely insufficient to preserve habitat near the present environmental limits inferred from the pteropods' physiological sensitivities. In the CCE specifically, the upper ocean is projected to warm by 1.6°–3°C by 2100 under RCP8.5, accompanied by a decrease of 0.37 to 0.75 in Ω_ar_ (Siedlecki et al., 2021). Thus, using the sensitivities in this work, we expect warming alone could exclude pteropods from most locations sampled during the WCOA cruises that have present temperatures of 9°–11°C: this covers much of the CCE. Projected Ω_ar_ decreases would also reduce habitat suitability by a similar degree as an additional 1°–3°C of warming, which could reduce habitat suitability even at cooler temperatures of 8°–10°C. The combination of CCE‐specific projections and environmental sensitivities suggests, barring substantial pteropod adaptive capacity and major emissions mitigation efforts, that *L. helicina* will be excluded from large portions of the CCE by 2100.

Beyond considering average changes in the surface ocean, OWA may change the distribution of *L. helicina* habitat with depth as well. All the species within the holoplanktonic gastropod community are epipelagic diel vertical migrators that experience large changes in their environment daily. This requires rapid metabolic acclimatization that is temperature dependent, with faster respiration in the warmer surface waters and a metabolic slowdown in the cold and corrosive depths of their daily migration (Smith & Teal, 1973). According to McLaren's hypothesis (McLaren, 1963, 2011), epipelagic migrators display uniform patterns of seeking lower temperature at deeper depths, an epipelagic adaptation that serves to conserve their energy (Oliver et al., 2020; Teal & Carey, 1967). This points to the importance of the vertical habitat as a refugia to limit surface thermal stress but may come at the cost of greater low‐Ω_ar_ stress at depth. The physiological impacts of both stressors were demonstrated through increased shell dissolution (Gardner et al., 2017; Lischka & Riebesell, 2012), increased respiration (Comeau et al., 2010; Hoshijima et al., 2017); and reduced precipitation (Comeau et al., 2010). Thus, compression of vertical habitat by each stressor will likely produce challenging trade‐offs for energy conservation by vertical migration (Oliver et al., 2020) and may lead to altered spatial and temporal distributions of vertical migrations (sensu Bednaršek and Ohman, 2015). In addition, increased metabolic demand from warming or ocean acidification might have synergistic implications related to food or oxygen limitation (e.g., Maas et al., 2012). Future research could investigate how multiple stressors affect the energy requirements and availability for homeostatic strategies, resulting in impaired physiology or mortality beyond the limitations we identified here.

We showed that our empirical HSI is consistent with experimental and empirical data at multiple scales of space and time. This agreement across varied environmental, population, and ecosystem conditions suggests that other factors might play a secondary role in defining *L. helicina* habitat suitability. Additionally, the relative genetic homogeneity of *L. helicina* across the Northern Hemisphere; i.e., across the North Pacific and North Atlantic (Bednaršek et al., 2021; Mekkes et al., 2021; Shimizu et al., 2018, 2021) may contribute to the similarity of physiological response across the data sets and approaches presented here. However, the temperature and Ω_ar_ stresses we identified are unlikely to be the only factors contributing to *L. helicina* distributions. When habitat is broadly favorable with respect to these properties, as expected in the wintertime CCE, other variables may become more important constraints on which habitat is occupied. Alternative hypotheses for primary drivers of pteropod distributions include variable food supply (Bednaršek et al., 2018; Appendix S1: Section S3) and physical advection along the coast. We briefly considered each by way of alternative HSI model formulations and present those results in Appendix S1. However, in brief, available alternative hypotheses were measurably worse at explaining the empirical observations than the simple two factor framework that we present here.

Our findings imply that predicted global and regional ocean warming and acidification could have stark implications for the future of *L. helicina* in the CCE, with similar repercussions for other *L. helicina* populations across the Northern Hemisphere. Thus, future modeling efforts should strive to consider OWA in predicting future outcomes for pteropod populations. The strong parallels between our empirical HSI results and the experimental treatment responses suggest that the model we present could be developed to project responses to future conditions, including more frequent or extreme marine heat wave events (Frölicher & Laufkötter, 2018; Oliver et al., 2018), enhanced upwelling, or intensifying anthropogenic ocean acidification. More broadly, tools such as HSIs can inform marine ecosystem vulnerability studies, local pollution impact assessments, climate projections, adaptation strategies, and near‐term management responses of sensitive indicators of global climate change.

## AUTHOR CONTRIBUTIONS

Nina Bednaršek wrote the paper with contributions from all co‐authors. Nina Bednaršek, Brendan R. Carter, Ryan M. McCabe, Richard A. Feely and Evan Howard conceived the research. Brendan R. Carter conducted the HSI analyses. Evan Howard analyzed OBIS data. Nina Bednaršek, Ryan M. McCabe, Brendan R. Carter, Richard A. Feely, Evan Howard, Zach Siegrist obtained and analyzed the data and provided visualizations. Richard A. Feely led the WCOA cruises and provided the water property data. Nina Bednaršek directed the zooplankton fieldwork and led the analyses of pteropod field efforts and experiments. Francisco P. Chavez provided carbonate chemistry data. Meredith Elliott, Jaime Jahncke, and Jennifer L. Fisher provided chemical and biological data.

## CONFLICT OF INTEREST

The authors declare no conflict of interest.

## Supporting information


Appendix S1

## Data Availability

In situ observations of pteropod presence and abundance collected in Bongo nets from NOAA ships in the Pacific Ocean for the NOAA West Coast Ocean Acidification cruises from 2011 to 2016 (Bednaršek & Feely, 2022) are available from the NOAA National Centers for Environmental Information (NCEI Accession 0251154) at https://doi.org/10.25921/459r-m724. Presence absence data for Limacina from NH‐25 from 2011 to 2016 from 2011 May 1 to 2016 June 30 (Fisher et al., 2022) are available from the NOAA National Centers for Environmental Information (NCEI Accession 0251631) at https://doi.org/10.25921/fx52-qe78.
