# Supplementary material for: Pelagic calcifiers face increased mortality and habitat loss with warming and ocean acidification
Source: Ecol Appl. 2022 Jul 27;32(7):e2674. doi: 10.1002/eap.2674 (PMC9786838; doi:10.1002/eap.2674)
Supplement: Supplementary file 1 — Appendix S1 [file EAP-32-e2674-s001.pdf]

Appendix S1

Nina Bednaršek[a,b*], Brendan R. Carter[c,d], Ryan M. McCabe[c,d], Richard A. Feely[c], Evan Howard[e], Francisco P. Chavez[f], Meredith Elliott[g], Jennifer L. Fisher[b], Jaime Jahncke[g], Zach Siegrist[i]

Pelagic calcifiers face increased mortality and habitat loss with warming and ocean acidification

Ecological Applications

Section S1

HSI model construction and validation

Several steps were taken to identify parameters and select the final model variants for the HSI. Preliminary exploration considered many potential combinations of parameters (*P* terms) and depth ranges for estimating habitat suitability, including *T*, salinity *S*, seawater spice (a measure of *T* and *S* variability at constant density and a powerful water mass tracer in the CCE; see 48), nitrate, nitrite, silicate, phosphate, surface chl-a, maximum and 100-integrated water column chl-a, pCO_2, pH, Ω_{ar}, Ω_{ar} saturation horizon depth, dissolved oxygen (DO), and 60% DO saturation depth. For HSI fitting, which requires measurements that are synoptic and co-located with the net-tows, we used measured surface chl-a or chl-fluorescence data and not values derived from satellite observations.

For each of these options, trials to evaluate model variants were conducted using water averaged over 20 depth surfaces ranging from the top 10 m to the top 200 m of the water column at 10 m increments. Values were comparable for deeper averages down to ~200 m, though deeper averages excluded stations with bottom depths that were shallower than 200 m. In two sets of trials, the training data were *L. helicina* presences/absences and seawater parameter distributions from either the 2011 or the 2016 WCOA cruise. A total of 40 trials were conducted with multiple parameter options. Any terms that were excluded from more than 95% of the models by the iterative X^2 test were removed from further consideration, leaving only *T*, *S*, Ω_{ar}, phosphate, and seawater spice. T was selected in >90% of trials, so it was included in all further tests.

The model validation metrics were used to shed light on the remaining terms. We validate the models using the following error metric, *E*:

$$E = \frac{\sum_{k=1}^{m} |\mathrm{HSI}_k - PA_k|}{m} 100\% \qquad \text{(Eqn. S1)}$$

where PA_k is a logical integer representing presence (1) or absence (0) of *L. helicina* in the *k*th plankton tow event out of m total events. *E* is a percentage, ranging from 0 to 100%, representing the average absolute difference between the model-estimated probability and the presence or absence found. A model that makes all predictions correctly with only 100% or 0% likelihoods would have an *E* value of 0%, while the *E* value would be 100% if all predictions were incorrect. Using error directly as a metric can be problematic when modeling lopsided distributions (i.e., when pteropods are either mostly present or mostly absent), so for this discussion we present the "skill improvement" (Eqn. S2), which presents *E* of the HSI model relative to the *E* for a constant probability model equaling the mean *PA* (E_{Constant}):

$$SI = 100\left(1 - \frac{E_{model}}{E_{constant}}\right)\% \qquad \text{(Eqn. S2)}$$

SI values for the various models constructed with temperature and other properties suggest the best models are those with *T* alongside either Ω_{ar} or *S* (Supplementary Table S4). *S* outperformed spice at 100 m, but we note that spice performed within 1 standard *SI* error (±7%) of *S* at most depths, and our omission of spice partially due to *S* being simpler and more frequently available in other datasets than spice. A model variant with all three variables (*T*, *S*, Ω_{ar}) included did not significantly outperform either the *T*-*S* or *T*-Ω_{ar} model variants. Phosphate, by contrast, performed poorly nearer the surface, and only nearly as well as Ω_{ar} below ~100 m. The empirical HSI is, except where noted, based on *T*-Ω_{ar} and fit to WCOA.

Section S2

Pteropod distributions from OBIS

Observations of *L. helicina* were obtained from the Ocean Biodiversity Information System (OBIS; https://obis.org/taxon/140223). We did not filter observations by animal size or life stage, as this was not noted for 87% of the data. Observations labeled as larval (4% of the total) were not spatially distinct from labeled adult and juvenile samples (5% and 4% of the total). Unlabeled observations were assumed to correspond predominantly to juvenile and adults, as most samples were collected with net mesh sizes greater than 0.2 mm. Of the 995 samples for which either life stage or size was available (all collected >46°N, and most north of the Arctic circle), 41% were identified as either adult or sizes >0.8 mm, 28% were identified as juvenile or had sizes between 0.2 and 0.8 mm, and the remaining 31% were identified as larvae, following that different life stages is not likely to alter the results quantitatively.

Datasets contributed to OBIS represent predominantly polar and subpolar sampling. We filtered out observations from the Gulf of St Lawrence; in situ hydrographic conditions at the time of pteropod sampling are poorly represented by World Ocean Atlas (WOA) and Global Ocean Data Analysis Project for Carbon gridded data product (GLODAP) climatological hydrographic observations we use in this work, because of the spatially and temporally dynamic estuarine

conditions in this region. Observations for which no spatially co-located hydrographic properties were available were also discarded from further analysis.

The vertical extent of net tows reported in OBIS vary widely across observations, reflecting varied study designs and samples of opportunity. In order to retain accurate depth ranges, we checked the maximum and minimum depths associated with each tow. We corrected reversed maximum and minimum depths in the database. Maximum depths that were recorded as being greater than local bathymetry was replaced with the bottom depth for pairing with hydrographic properties. When the minimum reported depth was 0 m and no maximum depth was reported, or only the bottom depth was reported without a minimum tow depth, observations were assumed to represent surface (0 m) samples. Observations without any reported depths (minimum or maximum) were assumed to correspond to surface samples as well. The maximum and minimum depth of each observation was assigned to the corresponding depth bins for WOA (102 standard depth levels) and GLODAP (33 depth levels). In the pelagic ocean, some observations were derived from net tows with too large a vertical range to constrain associated hydrographic conditions (e.g., a 1000 m vertical tow). For this work, we heuristically defined any tow exceeding 300 m vertical range between the maximum and minimum depth as insufficiently precise to constrain the true pteropod distribution. Because such extensive tows either included the ocean surface or had a minimum depth well below the ocean surface, we retained only the minimum depth from these tows as a potential constraint, and discarded the maximum depth.

After these data quality assurance and control steps, we extracted the monthly climatological temperatures and annual climatological aragonite saturation state for each location. To minimize biases due to higher temporal variability information in temperature than in Ω_{ar}, we averaged the monthly temperature climatology into an annual climatological average. We then binned the paired hydrographic data across the realized range of conditions for all observations (into 1°C by 0.2 Ω_{ar} bins) separately for maximum and minimum depth. Regardless of the actual pteropod observation depths, we additionally binned hydrographic data corresponding to the 0-100 m depth range representing near-surface conditions, for comparison with the CCE-specific HSI. In order to compare with the HSI and other relationships, for each observational depth set (maximum, minimum, 0-100 m), a slope was fit to the minimum Ω_{ar} value for which pteropod presence was observed at each temperature over 8-13°C range. This temperature range encompasses just the range of overlap between the CCE experiments and observations and the global database.

The analysis based on the maximum pteropod observation depths is presented in the 2.3 section. Using the maximum depth of observations maximizes the hydrographic spatial variability encompassed in the dataset and used to constrain the above relationships, while shallower depths are instead limited almost entirely by surface ocean (latitudinal) gradients. Using the maximum observed depths also results in the minimum Ω_{ar} over the evaluated temperature range deriving exclusively from observations at 90-760 m depth—in all cases well below the local seasonal

thermocline for each observation. At these depths, seasonal and interannual temperature variation is usually less than 0.5°C (Boyer et al. 2018, Roemmich & Gilson 2009). Carbonate system variability below the surface cannot be assessed with respect to the observation locations, but pelagic, subsurface Ω_{ar} is assumed to covary with temperature changes (directly through thermodynamic constraints or indirectly via community metabolism). Thus it is also expected to be substantially less variable than at the surface and suitably constrained for this analysis. Figures that are instead based on the minimum depth (predominantly 0 m) and 0-100 m depth ranges are provided in this Supplement for comparison (Fig. S2), however climatological hydrography may be inappropriate at these depths.

Section S3

Potential for food limitation or passive advection

We use chlorophyll-*a* and nutrient concentrations as proxies for the planktonic food availability for *L. helicina*. These can serve only as a gross proxy of potential productivity at the base of the food web rather than a specific tracer of the organisms these pteropods consume. Nonetheless, these are the only properties with sufficient spatial coverage of observations to evaluate the question of food limitation.

Chlorophyll-*a* fluorescence and surface chlorophyll-*a* concentrations were only measured during the 2016 cruise, and thus a chlorophyll-dependent HSI model could only be fit for this year. The HSI model based on *T* and chlorophyll-*a* performed substantially worse in that year ($R^2 = 0.37$ using 100 m integrated chlorophyll-*a*, R^2=0.42 with surface values) than did the 2016 T– Ω_{ar} model (R^2=1) or the presented mixed-year HSI ($R^2 = 0.67$ for all years of WCOA).

Macronutrient concentrations were measured in all years of WCOA cruises. Models that included nutrient concentrations also showed weaker predictive power and indicated those properties were anti-correlated with the likelihood of *L. helicina* presence. This anti-correlation is not conclusive because nutrient concentrations are not a measure of actual nutrient supply and resulting productivity in the surface ocean. Even so, we would have anticipated a positive correlation between nutrient concentrations and *L. helicina* presence if their nutrient-dependent phytoplanktonic food was a primary driver of pteropod distributions.

Alternately, Ω_{ar}—like salinity—may act as a water mass tracer revealing when equatorward advection passively brings *L. helicina* populations south along the coast from the poleward regions where the OBIS database suggests the organisms are more common. The HSI (presented in the Section 3.3) shows stronger correlations with *L. helicina* $\log_{10}$ abundances ($R^2 = 0.48$ for 2011, $R^2 = 0.37$ for 2016) than when the empirical HSI is fit to *T* and *S* alone ($R^2 = 0.36$ for 2011, $R^2 = 0.15$ for 2016, respectively). Finally, as noted in the section 3.5, the presented model has better *SI*

metrics for the timeseries data, even when using Ω_{ar} estimated from *T* and *S*, than the model fit directly to *T* and *S*.

Figure S1: Habitat suitability model outputs for *L. helicina* for each year of observations, depicted as (A–E) LRF (linearized HSI), (F–J) HSI (mid), and (K–O) % mortality. Abundance classes of *L. helicina* are shown as in Fig. 1.

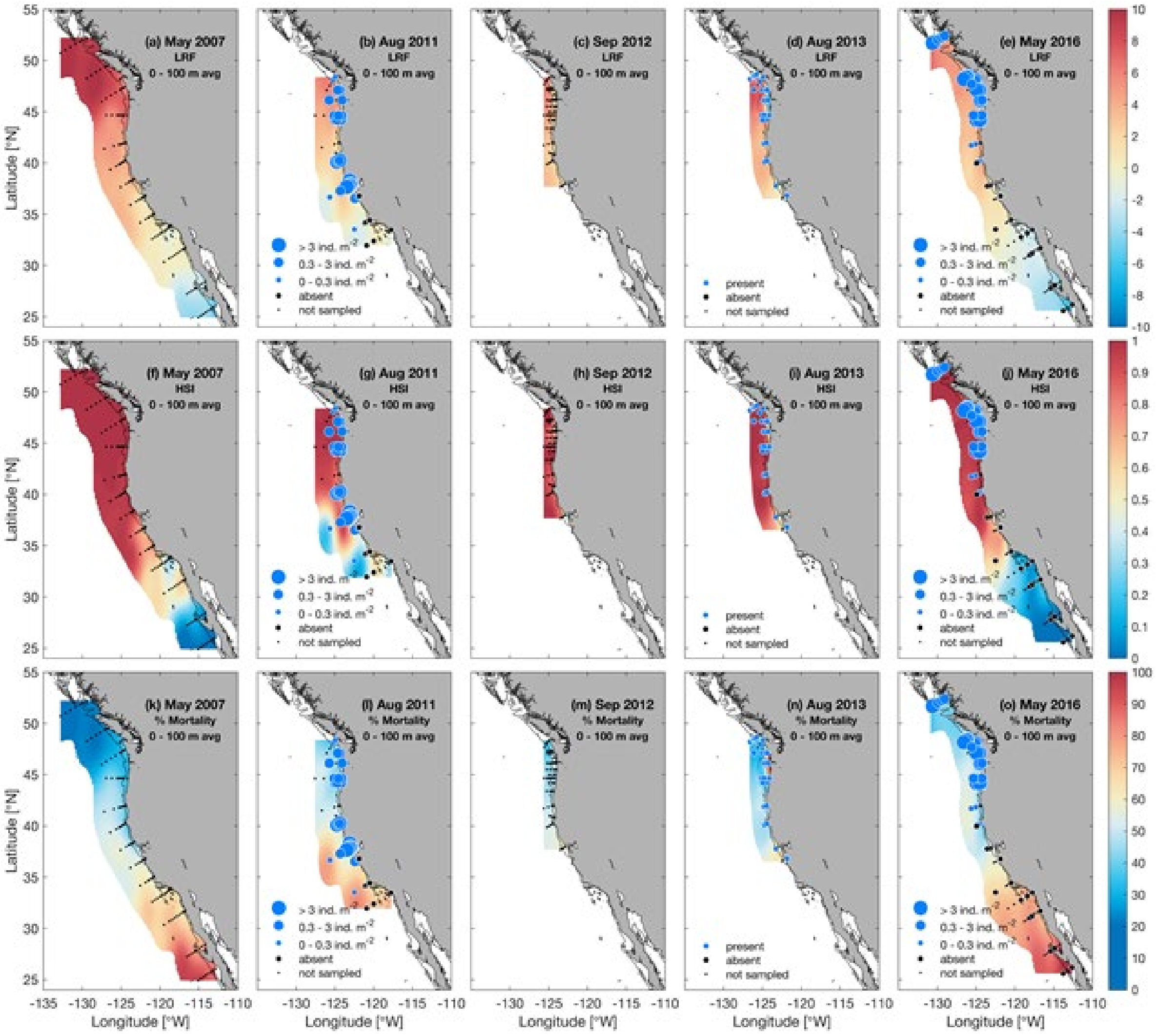

Figure S2: Global *Limacina helicina* presence (OBIS, shaded gray) with respect to annual mean temperature (WOA) and Ω_{ar} (GLODAP) at different depths, with lines representing equivalent relationships to those in Figure 6. In particular, the blue line represents the linear fit to the minimum observed Ω_{ar} over the overlapping temperature ranges between the WCOA and global datasets in each depth range, the black lines represent empirical relationships (WCOA with HSI=0.99, solid; abundance=0 relationship from Bednaršek et al. 2018, dashed), and the red line is the experimental mortality relationship equivalent to HSI=0.99). (a) Conditions at the minimum depth of the net tow ranges associated with each observation, often the ocean surface (blue line has $\Delta \Omega_{ar} / \Delta T = 0.21$, $R2=0.84$, $p=0.001$ for a null hypothesis of no correlation, for 8-16°C); and (b) Conditions averaged over 0-100 m depth regardless of the towed depths, comparable with the WCOA data treatment (blue line has $\Delta\Omega_{ar}/\Delta T=0.16$, $R2=0.88$, $p=0.002$, for 8-15°C). In contrast, Figure 6 plots conditions for only the maximum depth associated with each net tow generating a presence observation, which tends to be cooler and lower Ω_{ar} water compared to the surface.

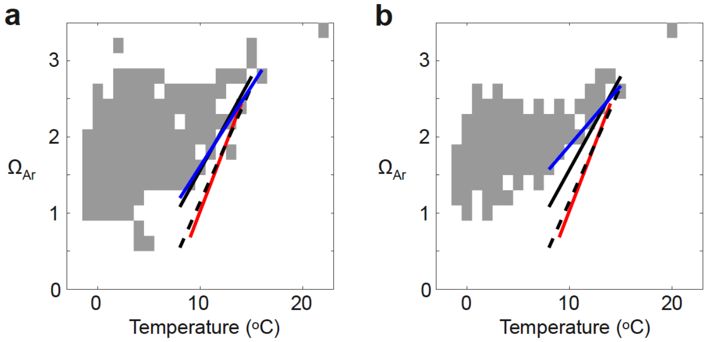

Table S1. Carbonate chemistry and auxiliary conditions with associated standard deviation in the multifactorial experimental setup.

parameters	HighT/high CO_2 High T/lowΩ_{ar}	High T/low CO_2 High T/high Ω_{ar}	Low T/high CO_2 Low T/low Ω_{ar}	Low T/lowCO_2 Low T/highΩ_{ar}
Temp (°C)	14.0±0.5	13.5±0.2	9.1±0.1	9.5±0.2
$p CO_2$ (µatm)	1200	400	1200	400
TSi (umol/kg^{-1})	2.2±1.3	2.2±1.3	2.2±1.3	2.2±1.3
TP (umol/kg^{-1})	0.28±0.11	0.28±0.11	0.28±0.11	0.28±0.11
salinity	31.1±0.30	31.1±0.30	31.1±0.30	31.1±0.30
pH	7.70±0.10	8.05±0.06	7.67±0.1	8.09±0.2
TA (umol/kg^{-1})	2296±323	2255±364	2398.1±326	2486±222
TC (umol/kg^{-1})	2254±304	2063±316	2361±402	2295±225
Ω_{ar}	0.94±0.25	2.20±0.6	0.92±0.3	2.25±0.70

Table S2. Upper: Treatment conditions aligned with the station of collection and experimental results of mortality (%) with standard deviation calculated from the raw data at different experimental conditions from different CTD stations. Twenty adult pteropod of *L. helicina* were included in each replicate, with six replicate per treatment. Lower: The results of Analyses of Variance (ANOVA) based on the experimental data with pCO_2 as a variable rather than Ω_{ar} (high CO_2 corresponds to low Ω_{ar}).

Treatment Description	Treatment Conditions	Sample size	% Dead
1	High T/high CO_2	240	100±0
2	High T/ low CO_2	240	64±25
3	Low T/high CO_2	160	48±26
4	Low T/low CO_2	160	17±9

	Df	SumSq	Mean Sq	F value	Pr(>F)
Temp	1	2.14509	2.14509	38.4536	4.67e-06 ***
pCO_2	1	0.88070	0.88070	15.7877	0.0007487 ***
interaction(temp:pCO_2)	1	0.01235	0.01235	0.2214	0.6430966
Residuals	20	1.11568	0.05578		

Table S3. Coefficients and R^2 terms for all linear functions (LF) discussed, with *P* terms surface 100 m integrated seawater properties (Fig. S3) and *α* values are their coefficients. *SI* is a model validation metric reflecting how well the model reproduced test data withheld from the training data set (higher is better). The mechanistic HSI is estimated from the incubation results as the amount survival (i.e., 100% minus Eqn. 5) is expected to exceed 33% as a LF (i.e., in Eqn. 4)*. The R^2 is the adjusted generalized R^2 term for the empirical HSIs. SI standard uncertainties are assessed at ±7%. ACCESS and Newport validation tests rely on synthetic Ω_{ar} distributions with significant uncertainties.

Eqn.	α_0	P_1	α_1	P_2	α_2	R^2	SI-WCOA	SI-ACCESS	SI-Newport
Mechanistic/Experimental	86.0	*T*	- 11.52	Ω_{ar}	32.75	0.78	80%*	25%	40%
Empirical with Ω_{ar}	13.49	*T*	-2.475	Ω_{ar}	10.10	0.67	55%	22%	38%
Empirical with salinity	163.75	*T*	-0.831	*S*	-4.612	0.68	50%	4%	39%

*As noted in the text, the choice of the 1/3rd survival threshold was based on the WCOA measurements. This choice is, in effect, using test data as training data in a non-quantitative sense, so SI statistics for this mechanistic model from WCOA data should not be considered a direct comparison to other WCOA SI values.

Table S4. *SI* metrics for empirical HSI models that use T and the listed parameters (in columns). The WCOA cruise year used for training data, the depth interval the properties are integrated over, and the number of viable measurements in the test data set are also indicated. Higher *SI* values indicate stronger predictive power in the test dataset, with 100 indicating a perfect model and 0 indicating the model does equivalently well to a constant-probability model. The uncertainty for these *SI* values was estimated with a bootstrapping approach at ± 7%.

			SI values (%)				
Training Data Year	Depth Interval	N (test data)	Mechanistic	Salinity	Spice	Phosphate	Ω_{ar}
2011	0 to 100 m	59	79	65	63	65	70
2016	0 to 100 m	48	80	35	23	20	39
